# Poisoning by Mushrooms

**Published:** 1874-10-01

**Authors:** N. S. Davis


### MEDICAL EXAMINER.

A

*Semi-Monthly Journal of Medical Sciences.*

EDITED BY N. S. DAVIS, M.D., AND F. H. DAVIS, M.D.

No. XIX. CHICAGO, OCT. 1, 1874. VOL. XV.

### Original Communications.

### POISONING BY MUSHROOMS.

By N. S. DAVIS, M.D.

ON the afternoon of Sept. 17th, 1874, a young man, attending the North-Western University, while out with two of his classmates gathering botanical specimens, plucked and ate mushrooms which he supposed to be edible. He had previously, during the same afternoon, eaten some sour grapes. They returned to their boarding place and while at the supper table the one who had eaten the mushroom began to be very sick. He had hardly more than time to leave the table when he began to vomit violently, with extreme distress in the epigastrium. The contents of the stomach were fully ejected but the paroxysms of vomiting and distress continued with great severity for three hours, during which he had some cramps in the muscles of the legs, occasional hiccough, skin and extremities cool, respiration twenty per minute, pulse one hundred, and extremely small and weak. After about three hours the bowels began to move, and he had, at intervals of half an hour, two or three copious serous discharges from the alimentary canal. These discharges were sufficient to fill a chamber vessel two-thirds full, were very thin and plainly tinged with blood; yet the pains in the abdomen were not very severe. Each intestinal evacuation was preceded and accompanied by severe cramps in the calves of the legs, and increased feebleness of the circulation, but the nausea and vomiting diminished. About six hours after the commencement of the attack all active evacuations ceased, and the patient began to have some sleep. During the last half of the night his stomach and bowels remained quiet and a moderate febrile reaction took place; yet, there was no stupor, no abdominal distension, and but little tenderness on pressure. He continued to have some redness and heat of skin, accompanied by moderate fullness of the pulse, slight dilatation of the pupils, dull headache and a sense of giddiness on assuming an erect position, all the next day. These symptoms passed away and he slept well the following night. The stomach exhibited slight morbid sensibility, with a feeling of general weakness for three or four days, when his recovery appeared to be complete.

I saw him in half an hour after the first active symptoms commenced. Learning that he had already vomited enough to fully evacuate the contents of his stomach, and that it had only been about two hours since he ate the *fungus*, I did not deem it necessary to give either emetics or cathartics, but required entire rest in the recumbent position, and to allay the extreme irritation of the gastric mucous membrane, gave him a teaspoonful of the following mixture every half hour.

℞ Carbolic Acid cryst. 8grs.
Glycerine, ℥ ss.
Camph. Tinct. Opii., ℥ ij.
Aqua Camph., ℥ ij.
Simple syrup, ℥ ss.
Mix.

After the bowels began to move and the pulse became very feeble he was ordered two tablespoonfuls of warm strong coffee between the doses of medicine.

These were all the remedies given, and how far they exerted any influence we leave every reader to judge for himself.

My object in placing this case on record is that it differs in several respects from the majority of cases of mushroom poisoning.

1st. The symptoms of irritation in this case commenced actively in two hours after the *fungus* was eaten, which is much sooner than in most of the cases on record.

2d. The symptoms were more like those of a purely irritant poison, acting directly on the mucous membrane of the stomach and bowels, while in most of the recorded cases symptoms of narcotism, such as stupor, delirium, &c., are prominent symptoms.

Since writing the above case, we learn that only a few days since two boys, at Oak Park, gathered what they supposed to be edible mushrooms, and caused the servant girl to cook them and participate in eating the same. They were all soon taken sick, and one of the boys, named Palmer Kellogg, twelve years of age, died. The other two are in a fair way to recover. We have not learned the particular symptoms exhibited in these cases.

A newspaper, in alluding to these cases, says the boys "picked a lot of *toadstools* thinking they were *mushrooms.*" It is proper to state that there is no reliable and easily recognized difference between *toadstools* or poisonous and non-poisonous mushrooms, and the only safe way is to let all these fungi alone.

---

TREATMENT OF ERYSIPELAS BY SUBCUTANEOUS INJECTION OF CARBOLIC ACID.—Dr. Aufrecht practiced injections of carbolic acid in doses of 0.60 centigramme in ten cases. Not only were the erysipelatous swelling and redness rapidly dissipated, but the temperature, pulse, and general health were remarkably improved.—*Centralblatt.*

